# Genomic characterization of highly pathogenic H5 avian influenza viruses from Alaska during 2022 provides evidence for genotype-specific trends of spatiotemporal and interspecies dissemination

**DOI:** 10.1080/22221751.2024.2406291

**Published:** 2024-09-17

**Authors:** Christina A. Ahlstrom, Mia Kim Torchetti, Julianna Lenoch, Kimberlee Beckmen, Megan Boldenow, Evan J. Buck, Bryan Daniels, Krista Dilione, Robert Gerlach, Kristina Lantz, Angela Matz, Rebecca L. Poulson, Laura C. Scott, Gay Sheffield, David Sinnett, David E. Stallknecht, Raphaela Stimmelmayr, Eric Taylor, Alison R. Williams, Andrew M. Ramey

**Affiliations:** a US Geological Survey, Alaska Science Center, Anchorage, AK, US; b US Department of Agriculture, National Veterinary Services Laboratories, Ames, IA, US; c US Department of Agriculture, APHIS Wildlife Service, National Wildlife Disease Program, Fort Collins, CO, US; d Alaska Department of Fish and Game, Fairbanks, AK, US; e US Fish and Wildlife Service, Anchorage, AK, US; f US Fish and Wildlife Service, Yukon Delta National Wildlife Refuge, Bethel, AK, US; g Alaska Department of Environmental Conservation, Anchorage, AK, US; h University of Georgia, Athens, GA, US; i Marine Advisory Program, Alaska Sea Grant, University of Alaska Fairbanks, Nome, AK, US; j US Department of Agriculture, APHIS Wildlife Service, National Wildlife Disease Program, Palmer, AK, US; k Department of Wildlife Management, North Slope Borough, Utqiagvik, AK, US; l Institute of Arctic Biology, University of Alaska Fairbanks, AK, US; m US Fish and Wildlife Service, Izembek National Wildlife Refuge, Cold Bay, AK, US

**Keywords:** Molecular epidemiology, bird flu, pathogen genomics, pathogen evolution, avian influenza

## Abstract

The ongoing panzootic of highly pathogenic H5 clade 2.3.4.4b avian influenza (HPAI) spread to North America in late 2021, with detections of HPAI viruses in Alaska beginning in April 2022. HPAI viruses have since spread across the state, affecting many species of wild birds as well as domestic poultry and wild mammals. To better understand the dissemination of HPAI viruses spatiotemporally and among hosts in Alaska and adjacent regions, we compared the genomes of 177 confirmed HPAI viruses detected in Alaska during April–December 2022. Results suggest multiple viral introductions into Alaska between November 2021 and August or September 2022, as well as dissemination to areas within and outside of the state. Viral genotypes differed in their spatiotemporal spread, likely influenced by timing of introductions relative to population immunity. We found evidence for dissemination of HPAI viruses between wild bird species, wild birds and domestic poultry, as well as wild birds and wild mammals. Continued monitoring for and genomic characterization of HPAI viruses in Alaska can improve our understanding of the evolution and dispersal of these economically costly and ecologically relevant pathogens.

## Introduction

Highly pathogenic avian influenza (HPAI) was historically considered a disease that principally affected domestic poultry [1]. Since 1996, this affliction has become a much broader One Health issue [2–4]. Through repeated spillover events occurring over more than two decades, HPAI viruses of the H5 goose/Guangdong (Gs/GD) lineage, and specifically those belonging to H5 clade 2.3.4.4b [5], have evolved such that they are readily maintained among wild birds [6,7]. Since approximately 2021, H5 Gs/GD 2.3.4.4b HPAI has expanded among wild and domestic birds at an unprecedented spatial scale, with numerous outbreak events reported on every continent with the single exception of Oceania [8–12]. This ongoing panzootic has resulted in historically large losses of domestic poultry and fur-bearing mammals [9,13–15], wild bird mortality events of such scale and breadth as to have potential implications to conservation efforts [16–18], as well as viral spillover to an unprecedented number and diversity of wild terrestrial and marine mammals [10,19–21].

Recent global reports have used information on disease manifestation and mortality among animals, as well as the detection and genomic characterization of viruses, to elucidate outbreak dynamics associated with H5 clade 2.3.4.4b HPAI viruses. For example, summaries of aerial surveys, mortality reports, productivity monitoring, and diagnostic testing provided evidence for large-scale die-offs and breeding colonies exhibiting poor reproductive success caused by HPAI among northern gannets (*Morus bassanus*), sandwich terns (*Thalasseus sandvicensis*), and other marine birds throughout the North Atlantic during 2021–2023 [16,18,22–25]. Reports of concurrent diagnostic sampling of stranded pinnipeds throughout the North Atlantic have provided evidence for viral spillover to gray (*Halichoerus grypus*) and harbour (*Phoca vitulina*) seals [20,26,27]. Phylogenetic analyses suggested that HPAI infection among North Atlantic marine birds and mammals involves multiple viral genotypes introduced through diverse pathways [20,24,28]. Serologic testing of live birds during or following these recent North Atlantic marine wildlife die-off events has provided evidence that some individuals survived infection, presumably mediated through an acquired immune response [16,18,28]. Less information has been reported on the serostatus of North Atlantic pinnipeds inhabiting areas affected by outbreak events [27].

In contrast to the extensive reports on H5 clade 2.3.4.4b HPAI viruses among wildlife inhabiting the North Atlantic, far less information has been reported on the infection status and impacts on wild birds and mammals inhabiting the North Pacific Basin [19,27]. Alaska is an area at the convergence of numerous migratory flyways for wild birds, inhabited by large populations of both wild terrestrial and marine mammals, and has no commercial poultry operations. There has been limited evidence to suggest large-scale mortality events associated with H5 clade 2.3.4.4b HPAI viruses in Alaska (Retrieved 18 April 2024, from the Wildlife Health Information Sharing Partnership event reporting system (https://whispers.usgs.gov)). However, sporadic detections of H5 clade 2.3.4.4b HPAI viruses among various taxa of wild birds and mammals in Alaska have been reported (https://www.aphis.usda.gov/livestock-poultry-disease/avian/avian-influenza/hpai-detections). Additionally, a summary of the occurrence of H5 clade 2.3.4.4b HPAI viral genotypes among wild and domestic birds throughout the United States during December 2021–April 2022 reported the occurrence of viruses of at least two genotypes among wild birds inhabiting Alaska, which included viruses introduced through migratory flyways from locations to the west (East Asia) and from the east (from Europe via the midcontinent of North America) [30]. Another nationwide summary of HPAI cases, this one focused on mammals, reported the detection of H5 clade 2.3.4.4b HPAI viruses from two red foxes (*Vulpes vulpes*) sampled in western and northwestern Alaska, again finding evidence for viral introductions into Alaska from both the west and the east [19]. A third, more regional investigation reported on the occurrence of H5 clade 2.3.4.4b HPAI viruses among hunter-harvested waterfowl in western Alaska and found evidence of viruses assigned to three hemagglutinin (HA) genotypes affecting hunter-harvested northern pintails (*Anas acuta*), an American wigeon (*Mareca americana*), and a cackling goose (*Branta hutchinsii*) [29]. Genetic evidence supported at least two viral introductions from East Asia as well as the previously described introduction from Europe via the mid-continent of North America. None of these three recent summary products, however, reported Alaska-wide information on the spatiotemporal or interspecies dissemination of H5 clade 2.3.4.4b HPAI viruses. In this study, we aimed to help fill data gaps regarding the recent occurrence of HPAI in the North Pacific Basin by assessing the genomic diversity, host range, and spatiotemporal dissemination of HPAI viruses detected throughout Alaska during 2022. Our objective was to identify trends that elucidate regional outbreak dynamics to inform future surveillance and response efforts.

## Materials and methods

Samples originating from state, federal, and tribal active and passive surveillance programmes in Alaska were submitted to National Animal Health Laboratory Network laboratories for influenza A testing. Active surveillance oropharyngeal/cloacal swab sampling of live birds was approved under U.S. Fish and Wildlife Service (USFWS) IACUC project 2021-004, U.S. Geological Survey (USGS) Federal Bird Banding Permit project 23984, and USFWS permit MB124992. Samples detected by influenza A PCR were sent to the U.S. Department of Agriculture Animal and Plant Health Inspection Service National Veterinary Services Laboratories for characterization including H5 clade 2.3.4.4b HPAI as previously described [30]. Genomic sequencing was attempted using RNA extracts from samples or virus isolates testing positive for Influenza A virus [31]. Raw sequencing reads were obtained from 228 samples originating from Alaska including those obtained from wild birds (*n* = 203), backyard poultry (*n* = 21), and mammals (*n* = 4).

Reads were assembled into viral genomes using a custom bioinformatic pipeline as previously described [29]. Data quality filters were incorporated as follows: gene segments were excluded from downstream analyses if they had less than 100% coverage or less than 100x average depth. Genomes were excluded from downstream analyses if the HA gene segment did not pass, or if fewer than three gene segments passed quality thresholds. All HA gene segment sequences were confirmed to have a polybasic cleavage site. Mutations associated with pathogenicity, receptor binding, replicative capacity, and transmission described in Suttie et al. [32] were queried using FluSurver (https://gisaid.org/database-features/flusurver-mutations-app/). Viral gene segments and genomes were assigned genotypes using GenoFLU (version 1.02; available at https://github.com/USDA-VS/GenoFLU). Gene segments with unique genotypes were compared to publicly available data using BLAST [33]. Information on the borough or census area (henceforth “borough”) of sample origin was used to assess and summarize the distribution of genome constellations throughout the state. All sequences have been submitted to GenBank under accession numbers PP801575-PP802914 and associated metadata are provided [34].

Bayesian phylogenetic analyses of concatenated genomes assigned to each genotype were conducted using BEAST v1.10.4 [35]. We included only genomes for which complete coding sequences for all eight gene segments passed quality thresholds. These analyses were supplemented with additional global sequences to estimate the time to most recent common ancestor (TMRCA) between HPAI viral sequences from Alaska and other regions for all genotypes detected in Alaska samples to gain inference on the general timing of introduction events. The TMRCA represents the first inferred transmission event in Alaska and is therefore a conservative estimate of the actual timing of introduction. To accomplish this, all H5N1 HPAI 2.3.4.4b genome sequences were downloaded from GISAID on October 30, 2023 (*n* = 6277) [36]. All complete viral genome sequences were assigned to genotypes using the GenoFLU tool and assigned to flyways (Supplementary Material). GISAID genomes matching genotypes found in Alaska during 2022 were retained and down sampled to optimize convergence using TempEst [37]: A3 (*n* = 58), A4 (*n* = 24), B2.1 (*n* = 30), B3.1 (*n* = 50), B3.2 (*n* = 119), B4.1 (*n* = 72). Additionally, a Bayesian maximum clade credibility (MCC) tree of the HA gene segment was generated. The top 100 BLAST hits for one query sequence representing each HA lineage were included in the analysis. Query sequences included A/bald eagle/Alaska/22-013001-001/2022, A/Canada goose/Alaska/22-013831-002/2022, and A/gadwall/Alaska/IZ22_0885/2022 for ea3, ea1, and ea4 HA lineages, respectively. Three independent Markov chains, each consisting of 200,000,000 steps and sampled every 20,000 steps, were each assessed for convergence and combined following a 10% burn-in. An uncorrelated relaxed clock model was used with a log-normal distribution and the Gaussian Markov random field Bayesian Skyride coalescent tree prior. The general time-reversible nucleotide substitution model with gamma-distributed rates was used, as determined using ModelTest in phangorn [38]. Maximum clade credibility trees were generated using TreeAnnotator (https://beast.community/treeannotator) and TMRCAs were separately assigned for each genotype to the common node from which all samples from Alaska diverged, based on both the whole genome analysis and the HA gene segment analysis. We assumed a single viral introduction into Alaska rather than multiple introductions of viruses of the same genotype. Trees were visualized using the package ggTree [39] in R version 4.2.0 [40]. A map of Alaska coloured by borough was created using the R package tigris [41].

## Results

### Multiple viral genotypes in Alaska

**Figure 1.**
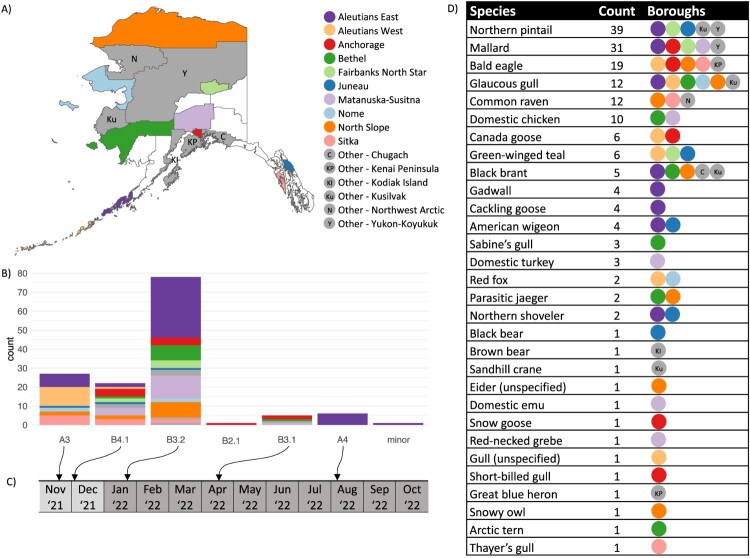
Spatiotemporal dissemination of H5 clade 2.3.4.4b highly pathogenic avian influenza viruses in Alaska in 2022. (A) Map of Alaska shaded by borough. Boroughs with fewer than five H5 clade 2.3.4.4b highly pathogenic avian influenza virus detections are coloured in grey and those without detections are not shaded. (B) Number of detections of viruses of each genotype, coloured by borough of detection (excluding the 38 samples that were unable to be genotyped). (C) Estimated median time to most recent common ancestor viruses of each genotype in Alaska (arrows). (D) 177 H5 clade 2.3.4.4b highly pathogenic avian influenza virus genomes obtained from each animal species and the boroughs from where they were detected.

Samples were collected between 26 April and 18 December 2022, though sampling date was unavailable for seven samples [34]. The location from which samples were collected varied through time, and nearly two-thirds (111/177) of samples yielding virus genomes were collected between 1 September and 18 December 2022 (Supplementary Figure 1). A total of 177 HPAI virus genomes originating from animals sampled across 16 Alaska boroughs passed our quality thresholds, including those from wild birds (*n* = 159), mammals (*n* = 4), and backyard poultry (*n* = 14; Figure 1) [34]. The most common species from which viral genomes were obtained in the final dataset were northern pintails (*n* = 39), mallards (*A. platyrhynchos*; *n* = 31), and bald eagles (*Haliaeetus leucocephalus*; *n* = 19; Figure 1). Waterfowl were the most common hosts of HPAI viruses in our dataset, represented by 58% (103/177) of samples from which viral genomes were obtained. When considering mammals, viral genomes were identified among samples collected from black bear (*Ursus americanus*), brown bear (*U. arctos*), and red fox (Figure 1) [34].

Regarding the HA gene segment, three lineages were detected, ea1, ea3, and ea4 (Table 1), as inferred using the GenoFLU tool. The ea1 HA lineage was most common, assigned to viral sequences originating from all 14 backyard poultry samples, two mammal samples (black bear, red fox), and 122 wild bird samples with origins from throughout the state (Table 2). The ea3 HA lineage was less common, identified among viruses originating from two mammals (brown bear, red fox) and 31 wild birds sampled in six boroughs. The ea4 HA lineage was least common, identified among viruses originating from only six wild bird samples, all of which were collected from the Aleutians East Borough (Table 1, Table 2).

**Table 1.** H5 clade 2.3.4.4b highly pathogenic avian influenza virus genotypes detected in Alaska wild birds, mammals, and backyard poultry, the time to the most recent common ancestor (TMRCA) of genotypes detected in Alaska, and the global migratory bird flyways inferred to be associated with dispersal at the time of analysis.

Genotype	PB2	PB1	PA	HA (H5)	NP	NA (N1)	M	NS	Whole genome TMRCA estimate	Flyways associated with dispersal to/from Alaska
A4	ea4	ea4	ea4	ea4	ea4	ea4	ea4	ea4	7 August 2022	East Asian-Australasian
A3	ea3	ea3	ea3	ea3	ea3	ea3	ea3	ea3	15 November 2021	East Atlantic*, Black Sea/Mediterranean*, East Asian-Australasian, Pacific, Central
B2.1	am1.2	ea1	ea1	ea1	am1.1	ea1	ea1	ea1	–	Pacific, Central, Mississippi, Atlantic
B4.1	am2.2	ea1	ea1	ea1	am1.3	ea1	ea1	ea1	1 December 2021	Pacific, Central, Mississippi, Atlantic
B3.1	am2.1	ea1	ea1	ea1	am1.4.1	ea1	ea1	ea1	14 April 2022	Pacific, Central, Mississippi
minor	am12	am11	ea1b	ea1	am1.3	ea1	ea1	am1.2	–	–
B3.2	am2.1	am1.2	ea1	ea1	am1.4.1	ea1	ea1	am1.1	22 January 2022	East Asian-Australasian, Pacific, Central, Mississippi, Atlantic

Note: Gene segments are shaded according to continental affiliation (Eurasian: dark grey, American: light grey).

*Flyways that do not overlap with Alaska.

**Table 2.** Number of H5 clade 2.3.4.4b highly pathogenic avian influenza virus genotypes detected in Alaska wild bird, mammal, and backyard poultry species (spp.) and location of detection (Alaska borough).

Genotype	# wild birds (spp. affected)*	# mammals (spp. affected)	# backyard poultry (spp. affected)	Location (Alaska borough)
A4	6 (AMWI, GADW, GWTE, NSHO)	0	0	Aleutians East
A3	26 (AMWI, BAEA, CORA, GLGU, NOPI, THGU)	1 (red fox)	0	Aleutians East, Aleutians West, Juneau, Nome, North Slope, Sitka
B2.1	1 (BAEA)	0	0	Anchorage
B4.1	18 (BAEA, BLBR, CAGO, eider sp., MALL, SBGU)	1 (black bear)	3 (chicken)	Aleutians East, Aleutians West, Anchorage, Bethel, Fairbanks North Star, Juneau, Kenai Peninsula, Matanuska-Susitna, North Slope, Sitka
B3.1	5 (BLBR, MALL, RNGR, SAGU)	0	0	Anchorage, Bethel, Chugach, Matanuska-Susitna
minor	1 (NOPI)	0	0	Aleutians East
B3.2	72 (AMWI, ARTE, BAEA, BLBR, CAGO, CCGO, CORA, GADW, GLGU, GWTE, MALL, NOPI, PAJA, SACR, SAGU, SNGO, SNOW)	1 (red fox)	5 (chicken, domestic turkey)	Aleutians East, Anchorage, Bethel, Fairbanks North Star, Juneau, Kusilvak, Matanuska-Susitna, Nome, North Slope, Northwest Arctic, Sitka, Yukon-Koyukuk

*AMWI = American wigeon (*Mareca americana*), ARTE = Arctic tern (*Sterna paradisaea*), BAEA = bald eagle (*Haliaeetus leucocephalus*), BLBR = black brant (*Branta bernicla nigricans*), CORA = common raven (*Corvus corax*), CCGO = cackling goose (*B. hutchinsii*), CAGO = Canada goose (*B. canadensis*), GADW = gadwall (*M. strepera*), GLGU = glaucous gull (*Larus hyperboreus*), GWTE = green-winged teal (*Anas crecca*), MALL = mallard (*A. platyrhynchos*), NOPI = northern pintail (*A. acuta*), NSHO = northern shoveller (*Spatula clypeata*), PAJA = parasitic jaeger (*Stercorarius parasiticus*), RNGR = red-necked grebe (*Podiceps grisegena*), SACR = sandhill crane (*Antigone canadensis*), SAGU = Sabine's gull (*Xema sabini*), SBGU = short-billed gull (*L. canus*), SNGO = snow goose (*Anser caerulescens*), SNOW = snowy owl (*Bubo scandiacus*), and THGU = Thayer's gull (*L. glaucoides thayeri*).

When considering concatenated whole genome sequences, we identified seven unique genotypes (A3, A4, B2.1, B3.1, B3.2, B4.1, and a minor genotype), two of which were comprised of fully Eurasian lineage gene segments (A3 and A4), and the remaining comprised of gene segments of mixed continental affiliation including two to four North American lineage gene segments (PB2, PB1, NP, and/or NS) (Table 1). Thirty-seven samples did not yield complete genomes (i.e. missing ≥ 1 gene segment of sufficient quality) and were therefore excluded from whole genome genotype assignment.

MCC phylogenetic trees and TMRCA estimates were separately generated based on (i) all HA gene sequences and (ii) the concatenated whole genome of each independent genotype. Based on the HA gene segment, one sample (A/common_raven/Alaska/22−021856−001/2022), identified as genotype B3.2, clustered with B3.1 virus genotypes, likely indicating emergence via reassortment (Supplementary Figure 2). TMRCA estimates based on the whole genome suggest earlier introductions than do estimates based only on the HA gene segment. However, the order in which viruses were inferred to be introduced in Alaska was identical based on both methods.

### Spatiotemporal dissemination within Alaska and associated flyways

#### Genotypes exhibiting limited dissemination within Alaska

Viruses of Genotype A4 were identified in the Aleutians East Borough in southwestern Alaska (Figure 1) and, based on publicly available data, had only previously been detected in the East Asian-Australasian Flyway (Table 1). Viruses of this genotype consist of wholly Eurasian lineage gene segment sequences, including HA lineage ea4, and were identified in six wild bird samples (Table 2), all of which were from waterfowl and collected on the same day (14 October 2022). Phylogenetic analysis of whole genome sequences indicated closest similarity to viruses sampled from waterfowl and chickens in Japan (Supplementary Figure 2). This genotype was estimated to be most recently introduced into Alaska with a TMRCA based on the concatenation of all eight gene segments of 17 August 2022 (95% CI: 15 July 2022–14 September 2022) (Figure 1; Supplementary Figure 3).

The single virus assigned to a minor genotype was isolated from a northern pintail sample also collected in the Aleutians East Borough (Figure 1) and, at the time of analysis, was the only recorded detection of this genotype globally. This genotype has a PB1 gene segment of the am11 lineage (Table 1), which was not identified in any other HPAI 2.3.4.4b genome in our search within the GISAID database. Based on BLAST analysis, this gene segment was most closely related (98.77% identity) to a H5N2 low pathogenic avian influenza virus isolated from a northern shoveller (*Spatula clypeata*) also sampled in the Aleutians East Borough (A/northern shoveller/Alaska/17-004479-3/2016(H5N2)), suggesting this genotype may have emerged through a reassortment event in this region. Its HA gene segment most closely resembles HA sequences of the B4.1 genotype (Supplemental Figure 2). A TMRCA could not be estimated for this viral genotype given its single detection; however, it was identified from a sample collected on 28 September 2022.

A virus of Genotype B2.1 was identified in a single bald eagle sample collected in the Anchorage borough on 9 May 2022 (Table 2), and viruses of this genotype have previously been detected in all four North American flyways. Of the seven genotypes detected in Alaska in 2022, Genotype B2.1 was the most commonly reported genotype in GISAID at the time of analysis (Supplementary Material). Since this was a single detection, a TMRCA was not estimated.

Viruses of Genotype B3.1 were identified in five wild bird samples, four from waterfowl and one from a gull, collected from four Alaska boroughs (Table 2, Figure 1) during a two-week period 27 May–8 June 2022. Viruses of the same genotype had previously been detected in the Pacific, Central, and Mississippi Flyways (Table 1); and based on the concatenation all eight gene segments, Alaska viruses shared an estimated TMRCA of 14 April 2022 (95% CI: 1 April 2022–27 April 2022) (Figure 1; Supplementary Figure 4).

#### Genotypes exhibiting widespread dissemination within Alaska

Viruses of Genotype A3 were identified in samples from 26 wild birds and one mammal collected from six Alaska boroughs (Table 2, Figure 1). Viruses of this genotype consisted entirely of Eurasian-lineage gene segments (Table 1). Based on BLAST analyses of the HA gene segment, Genotype A3 viruses from Alaska were most closely related to those from chicken and crows sampled in Japan. The earliest detections of Genotype A3 viruses in Alaska were from the Aleutians West Borough, suggesting introduction to Alaska via the East Asian-Australasian Flyway (Supplementary Figure 5). However, this genotype has been reported widely across many flyways, including the Central and Pacific Flyways of North America and the East Atlantic, Black Sea/Mediterranean, and East Asian-Australasian Flyways of Asia and Europe (Supplementary Material). The TMRCA of Alaska viruses, based on the concatenation of all eight gene segments, was estimated to be 15 November 2021 (95% CI: 13 October 2021–9 December 2021), suggesting it was the first HPAI viral genotype to be introduced into the state (Figure 1; Supplementary Figure 5).

Genotype B4.1 viruses were identified in 18 wild bird, one mammal (black bear), and three backyard poultry samples collected from 10 Alaska boroughs (Table 2, Figure 1). Genotype B4.1 viruses have previously been detected in the Atlantic, Mississippi, Central, and Pacific Flyways. Viruses from Alaska shared an estimated TMRCA, based on the concatenation of all eight gene segments, of 1 December 2021 (95% CI: 15 September 2021–23 January 2022) (Figure 1; Supplementary Figure 6).

Viruses of Genotype B3.2 were the most frequently detected in Alaska during 2022, having been identified among 72 wild bird, one mammal (red fox), and five backyard poultry samples collected in 12 Alaska boroughs (Table 2, Figure 1). Viruses of this genotype have previously been reported in all four North American flyways as well as the East Asia-Australasian Flyway. Alaska viruses of this genotype shared an estimated TMRCA, based on the concatenation of all eight gene segments, of 22 January 2022 (95% CI: 30 December 2021–13 February 2022) (Figure 1; Supplementary Figure 7).

### Dissemination among species

Of the five viral genotypes detected in more than one sample in Alaska (A4, A3, B4.1, B3.1, and B3.2), all were identified among samples from multiple species of wild birds, including more than one waterfowl species (Table 2). Viruses assigned to three genotypes with widespread spatiotemporal dissemination in Alaska during 2022 (A3, B4.1, and B3.2), which were also the most common viral genotypes by number of detections, were identified among more diverse host sources (Table 2). More specifically, all three spatiotemporally widespread genotypes were detected from wild bird samples representing multiple guilds of birds, such as waterfowl, raptors, and seabirds. Viruses of these genotypes were also identified among samples collected from wild mammals. Viruses of genotypes B4.1 and B3.2 were also identified from backyard poultry (Table 2). Ten mutations associated with pathogenicity, receptor binding, replicative capacity, or transmission were identified in at least one virus genome (Supplementary Table 1) [34]. Only one genome, obtained from a black bear sampled in the Juneau borough, harboured a mutation that was unique to mammals (i.e. PB2 E627 K was not found in avian samples).

Viruses of four genotypes (A4, B2.1, B3.1, and minor) exhibiting limited spatiotemporal dissemination in Alaska during 2022, which were also less common by number of detections, were identified only among samples collected from wild birds. Genotype B2.1, identified from a single sample collected from a bald eagle, was the only viral genotype not identified from at least one waterfowl sample in Alaska during 2022. In contrast, viruses of Genotype A4 were detected exclusively among waterfowl samples (Table 2). Viruses of Genotype B3.1 exhibited broader taxonomic host diversity, having been identified among samples collected from black brant *(Branta bernicla nigricans*), mallard, red-necked grebe (*Podiceps grisegena*), and Sabine’s gull (*Xema sabini*; Table 2) [34].

## Discussion

We analyzed 177 H5 clade 2.3.4.4b HPAI viruses originating from Alaska wild birds, backyard poultry, and mammals in 2022. Our results provide new insights into the spatiotemporal and interspecies dissemination of HPAI viruses in the North Pacific Basin. For example, our analysis provides genomic evidence for the occurrence of seven genotypes of H5 clade 2.3.4.4b HPAI viruses among wild and domestic animals inhabiting Alaska during 2022, including six genotypes likely introduced to Alaska from adjacent regions in East Asia or elsewhere in North America during November 2021–August 2022, as well as one viral genotype that may represent a locally emergent reassortant virus. Viruses of the B2, B3 and B4 genotypes were previously estimated to originate from reassortment events in North Dakota and Montana, whereas both A3 and A4 were likely introduced into Alaska from East Asia [29,30]. Our data suggest four viral genotypes exhibited limited spatiotemporal spread throughout Alaska during 2022, whereas viruses of three other genotypes were widespread both within Alaska as well as throughout adjacent regions. A similar trend was observed in British Columbia, Canada, with some genetic clusters of viruses more widespread over space and time than others [42]. Additionally, our data demonstrate widespread occurrence of HPAI viruses among waterfowl and dissemination to other taxa, including raptors, seabirds, domestic poultry, and wild mammals. This information contributes to the documentation of the unprecedented expansion of the ongoing HPAI panzootic throughout an ecologically important area of the North Pacific Basin region and provides insights into epidemiological trends that may ultimately prove useful for understanding future outbreak dynamics.

Viruses of four genotypes (A4, B2.1, B3.1, and the minor genotype) exhibited limited spatiotemporal dissemination in Alaska and were also found exclusively in wild birds. Reasons for this finding are not entirely clear but could be related to differences in infectivity, transmissibility, or pathobiology among H5 clade 2.3.4.4b viruses of different genotypes when considering diverse animal hosts inhabiting Alaska. Alternatively, this finding could be a function of the timing of viral introduction relative to population immunity. Per the former, previous studies have identified considerable differences in infectivity, transmissibility, and/or pathobiology of different H5 clade 2.3.4.4 viruses when comparing: (1) among experimentally infected chickens [43], (2) among chickens, turkeys, and ducks [6,44], or (3) among various avian and mammalian cell lines and model organisms [45,46]. Thus, it is plausible that some or all the viral genotypes exhibiting limited spatiotemporal dissemination were poorly adapted to avian and/or mammalian hosts present in Alaska during 2022. However, Genotype B2.1 was only detected in a single sample in Alaska, despite having been detected widely throughout the continental United States and Canada. Furthermore, all four of the viral genotypes exhibiting limited dissemination in Alaska during 2022 shared later estimates of TMRCA with viruses affecting wild birds in other regions, and they were initially detected in Alaska later, as compared to the three viral genotypes that were inferred to be more widespread. These findings suggest that the timing of viral introduction events into Alaska relative to pre-existing population immunity may, at least partially, explain differences in spatiotemporal dissemination.

Viruses of three genotypes exhibiting widespread spatiotemporal dissemination in Alaska during 2022 (A3, B4.1, and B3.2) may have been introduced into comparatively more immunologically naïve animal populations as compared to those described as being less commonly detected, facilitating more common and geographically widespread occurrence, at least early in the regional epidemic. Previously published summaries provide extensive evidence that numerous species of waterfowl and seabirds are commonly infected by low pathogenic influenza viruses in Alaska [47,48], which may have provided some protection against infection and disease [49], though antibodies specific to H5 clade 2.3.4.4b HPAI viruses were likely uncommon among these birds prior to late 2021 [50]. Furthermore, detectable antibodies to influenza viruses were also likely relatively uncommon among wild mammals inhabiting Alaska, such as bears and foxes, prior to the ongoing panzootic [51,52]. Thus, the H5 clade 2.3.4.4b viruses initially introduced into Alaska may have had to overcome only low barriers to infection, at least from a population immunity perspective, as well as being antigenically distinct from North American viruses. Per estimates provided through phylogenetic analyses presented in this study, viruses of Genotype A3 may have been the first H5 clade 2.3.4.4b HPAI viruses to be introduced to Alaska, presumably from East Asia and approximately during November 2021. Genotypes B4.1 and B3.2 presumably arrived sometime relatively shortly thereafter from elsewhere in North America. All these genotypes are reported to be spatiotemporally widespread, either throughout substantial portions of North America (B4.1) or across international migratory bird flyways crossing multiple continents (A3 and B3.2), attesting to their viral fitness among avian hosts. Viruses of the remaining, less spatiotemporally widespread, genotypes (A4, B2.1, B3.1, and the minor genotype) were all estimated to have been introduced into, or were detected later in, Alaska. These viruses may have, therefore, been introduced following the establishment of a population of recovered animals with antibodies protective against infection with H5 clade 2.3.4.4b HPAI viruses. Collectively, this scenario suggests that information on population immunity, the timing of viral introductions, and the occurrence of viruses in populations with spatiotemporal overlap in distribution may all provide value towards understanding, and predicting, epidemiological patterns of H5 clade 2.3.4.4b HPAI virus dissemination among wild and domestic animal populations.

There are several important sampling biases in our data that could have influenced results. First, samples were largely collected passively (i.e. opportunistically from sick animals or carcasses) for most animal species except waterfowl. Passive collection of carcasses was biased towards certain species (e.g. bald eagles) due to their visibility and protection status, whereas other species (e.g. landbirds) were rarely sampled due to lower detection probability of carcasses. Samples from waterfowl were disproportionally collected actively (i.e. from live or recently harvested birds that were apparently healthy) as part of interagency surveillance programmes that leveraged banding programmes or the annual sport harvest in late summer and autumn as part of sampling protocols. These samples may provide a more unbiased assessment of circulating viruses in a reservoir host, though were disproportionally collected in a single borough (Aleutians East Borough) [53]. Sequences were not differentiated as originating from samples collected from hunter-harvested animals, animals sampled through monitoring efforts (e.g. banding), or animals that were collected from mortality events. Also, sampling efforts in Alaska were not uniform throughout space nor time, as active surveillance was concentrated in the second half of the year. Many areas of Alaska are not readily accessible by road or commercial airline and have extremely low population density; therefore, samples of suitable quality for diagnostic testing were disproportionally collected near larger, road-accessible or commercial airline-serviced hub communities. Furthermore, given limitations on resources, samples from species that had previously been unknown to harbour H5 clade 2.3.4.4b HPAI viruses in Alaska, or that originated from areas where such viruses had not been previously identified, were often prioritized for sample collection and testing in passive surveillance efforts. Finally, when estimating TMRCA for viruses of different genotypes in Alaska with those from elsewhere, we assumed a single viral introduction into Alaska rather than multiple introductions of viruses of the same genotype, which we recognize as a probable epidemiological simplification. While TMRCA is not directly linked to virus introduction, it is our best proxy for estimating the approximate timeframe and order of introduction of distinct virus genotypes. It is unknown how these various biases individually or collectively influenced our results, though we recognize that they may have bearing on the results and subsequent inference.

In summary, we used all genomic sequence information available for H5 clade 2.3.4.4b HPAI viruses from animals sampled in Alaska during 2022 to determine that at least seven viral genotypes affected diverse wild birds, backyard poultry, and wild mammals. Five genotypes were detected in the Aleutians East Borough and six genotypes were detected in waterfowl, suggesting this region and these taxa may be effective targets for future surveillance efforts aiming to identify viral genotypes introduced into Alaska and maintained among wildlife. Evidence suggests that viruses of three genotypes (A3, B4.1, and B3.2) were widespread throughout the state and among diverse hosts, likely facilitated by earlier introduction into the region as compared to genotypic viral counterparts. Viruses of four viral genotypes (A4, the minor genotype, B2.1, B3.1) were less spatially widespread and detected only among wild birds, which may have been influenced by later introduction to the region when population immunity may have been comparatively higher. These results suggest information on the timing of viral introductions and pre-existing population immunity may be useful in understanding, and perhaps predicting, patterns of H5 clade 2.3.4.4b HPAI virus dissemination among wild and domestic animal populations.

## Supplementary Material

Ahlstrom et al HPAI in AK supplementary table 1.xlsx

Ahlstrom et al HPAI in AK Supplementary Materials.xlsx

Ahlstrom et al HPAI in AK supplemental figures revised.pdf
