# Supplementary material for: Genomic characterization of highly pathogenic H5 avian influenza viruses from Alaska during 2022 provides evidence for genotype-specific trends of spatiotemporal and interspecies dissemination
Source: Emerg Microbes Infect. 2024 Sep 17;13(1):2406291. doi: 10.1080/22221751.2024.2406291 (PMC11443546; doi:10.1080/22221751.2024.2406291)
Supplement: Ahlstrom et al HPAI in AK supplemental figures revised.pdf [file TEMI_A_2406291_SM2373.pdf]

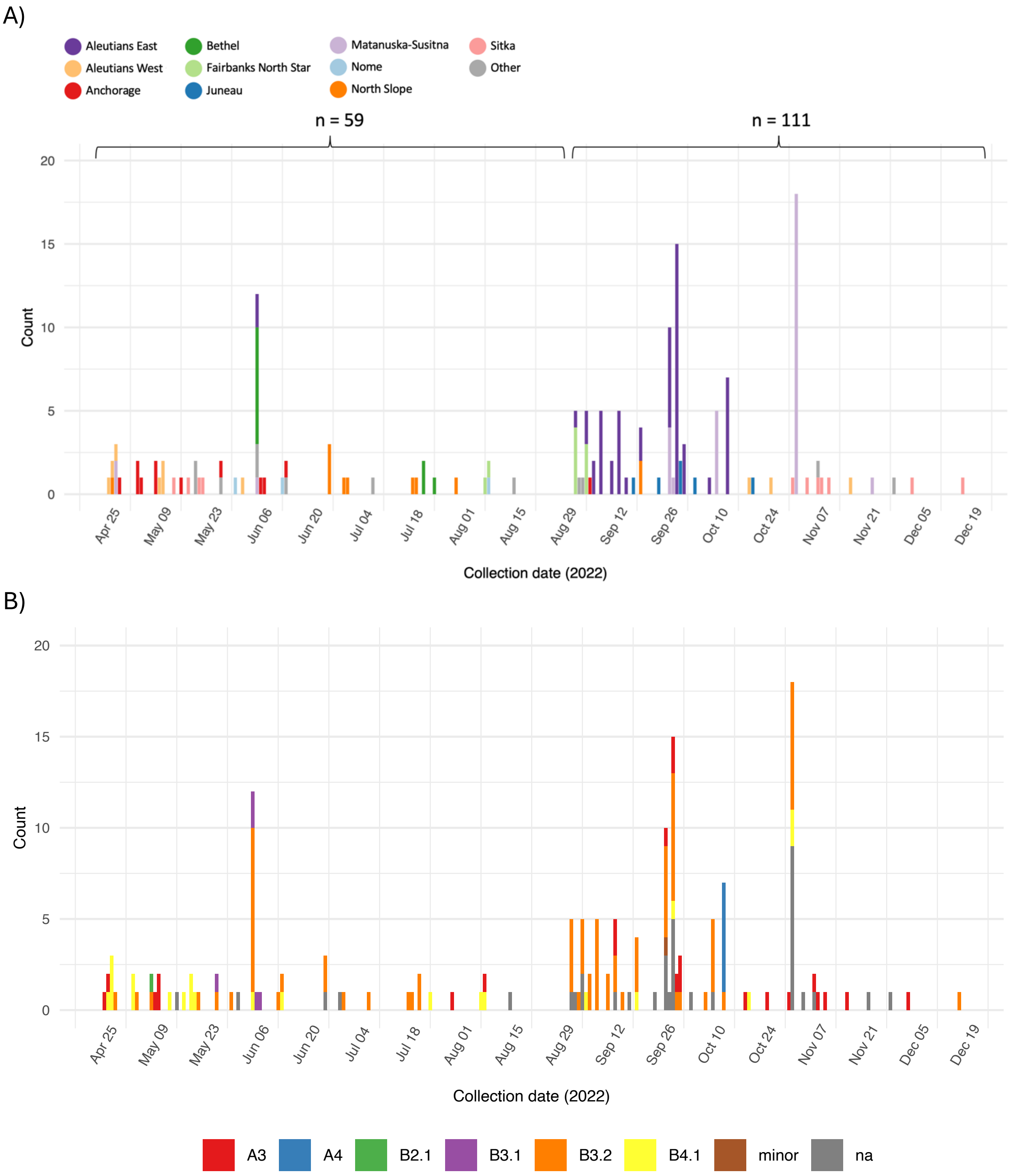

Supplementary Figure 1 Collection dates in 2022 of samples from which H5 clade 2.3.4.4b highly pathogenic avian influenza virus genomes were obtained. Colors represent A) sampling location (Alaska boroughs) and B) Genotypes (na indicates that a genotype was unable to be assigned).

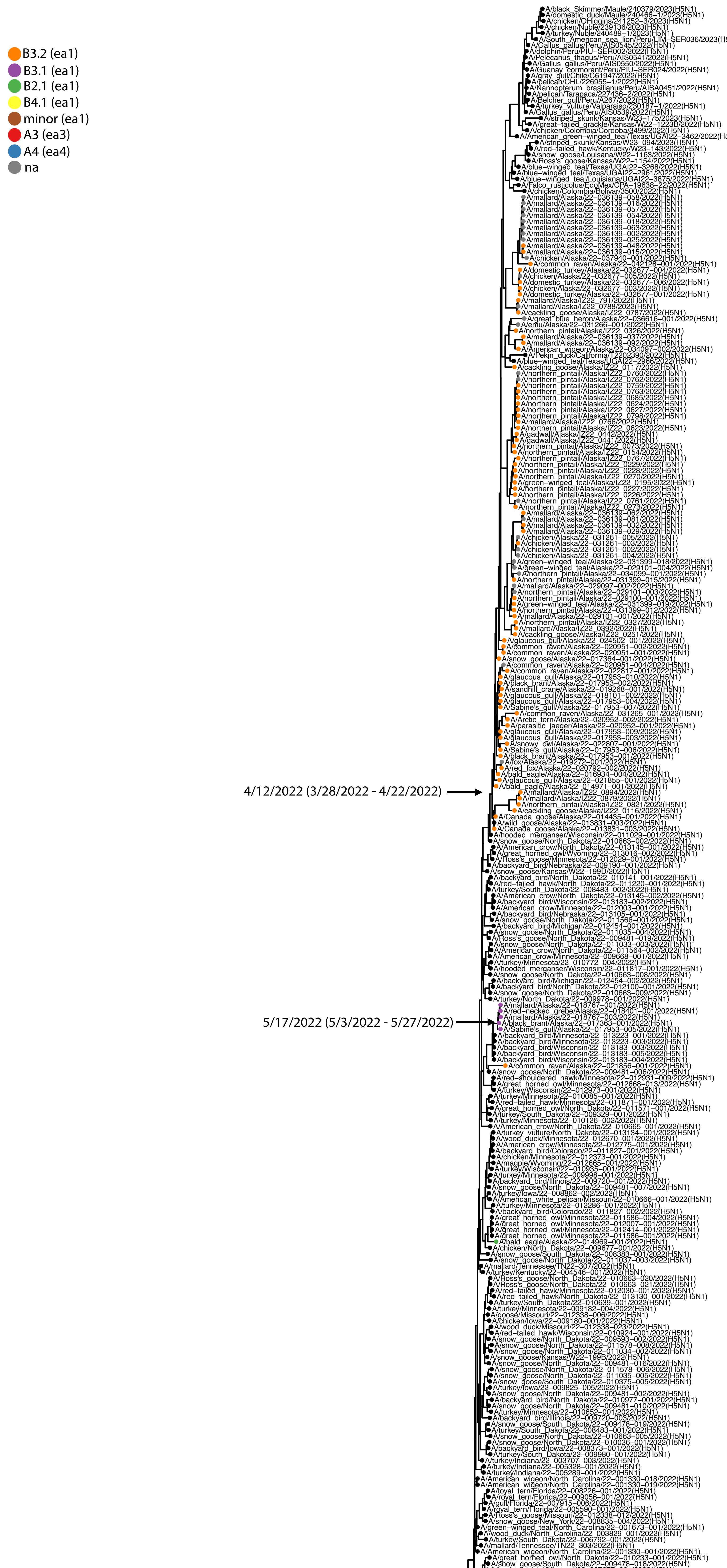

Supplementary Figure 2 Maximum clade credibility tree of H5 clade 2.3.4.4 highly pathogenic avian influenza virus sequences based on the HA gene segment from Alaska (colored tips) and elsewhere. Tips are colored according to genotype. Time to the most recent common ancestor nodes are indicated for each genotype with an arrow indicating the node from which all Alaska viruses of that genotype diverged. 95% highest posterior density intervals are presented in parenthesis.

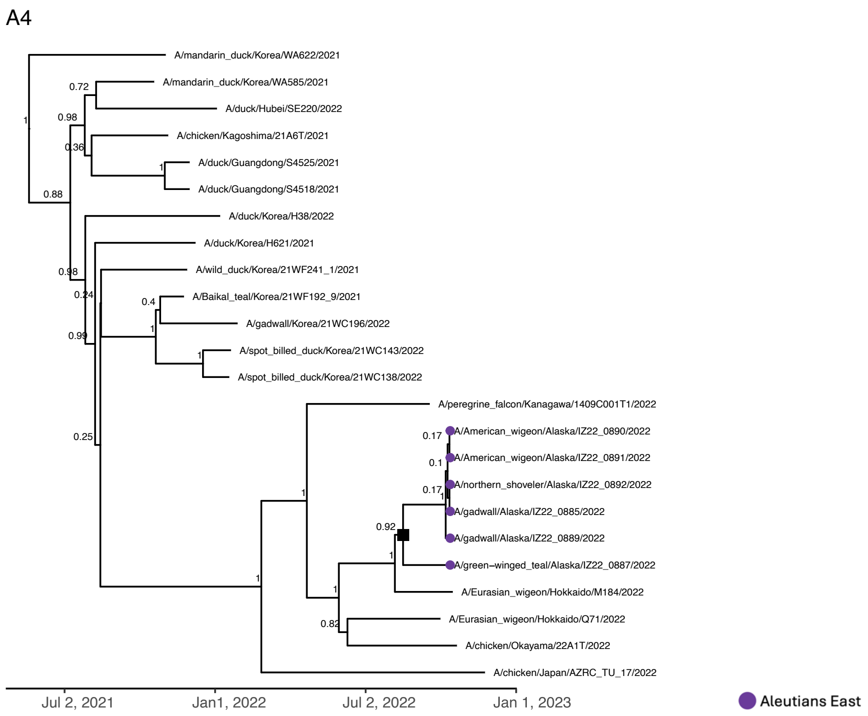

Supplementary Figure 3 Maximum clade credibility tree of Genotype A4 H5 clade 2.3.4.4b highly pathogenic avian influenza virus whole genomes from Alaska (colored tips) and elsewhere. A black square indicates the node from which the time to most recent common ancestor was estimated. Node labels indicate posterior probabilities.

Aleutians East

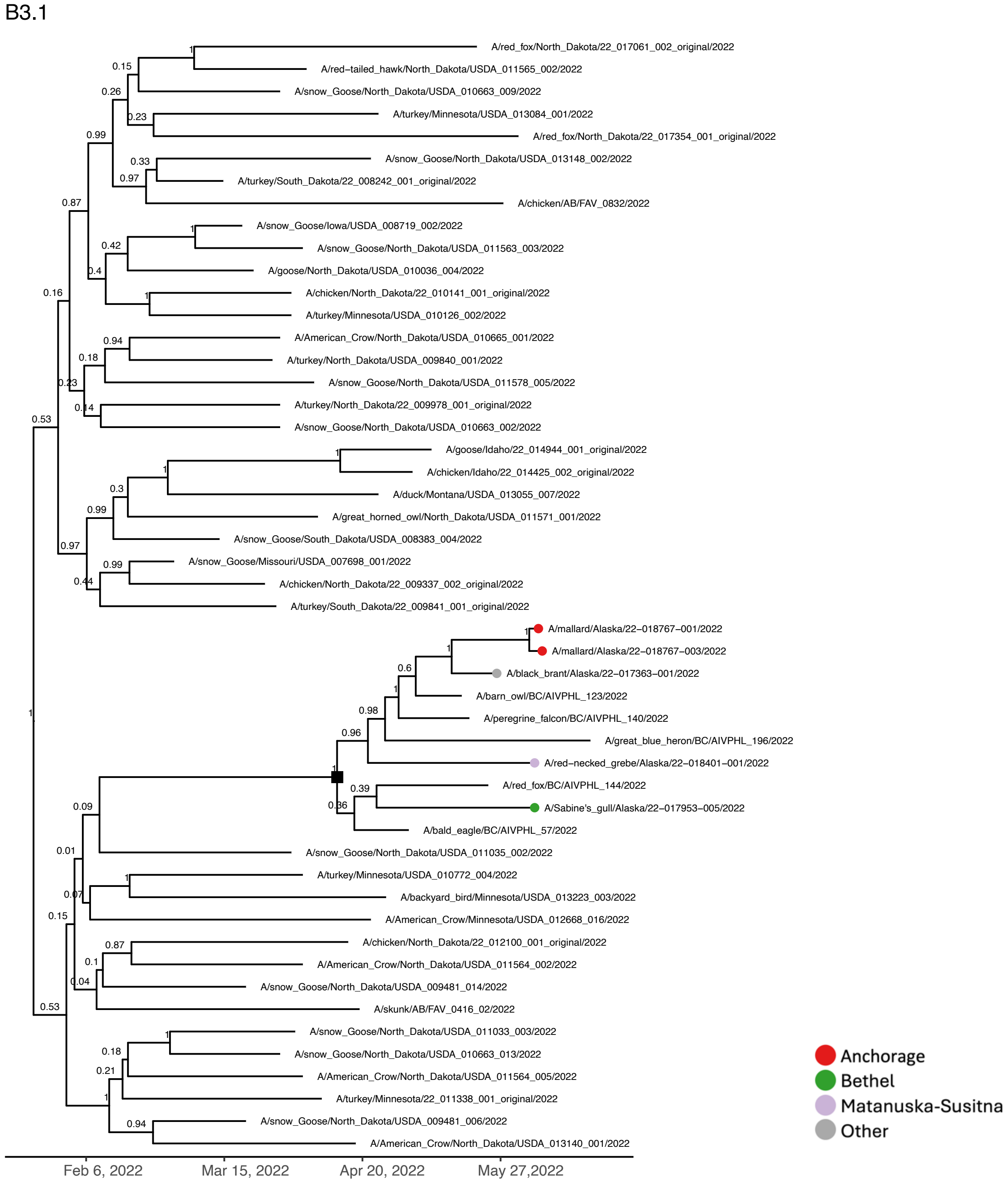

Supplementary Figure 4 Maximum clade credibility tree of Genotype B3.1 H5 clade 2.3.4.4b highly pathogenic avian influenza virus whole genomes from Alaska (colored tips) and elsewhere. A black square indicates the node from which the time to most recent common ancestor was estimated. Node labels indicate posterior probabilities.

A3

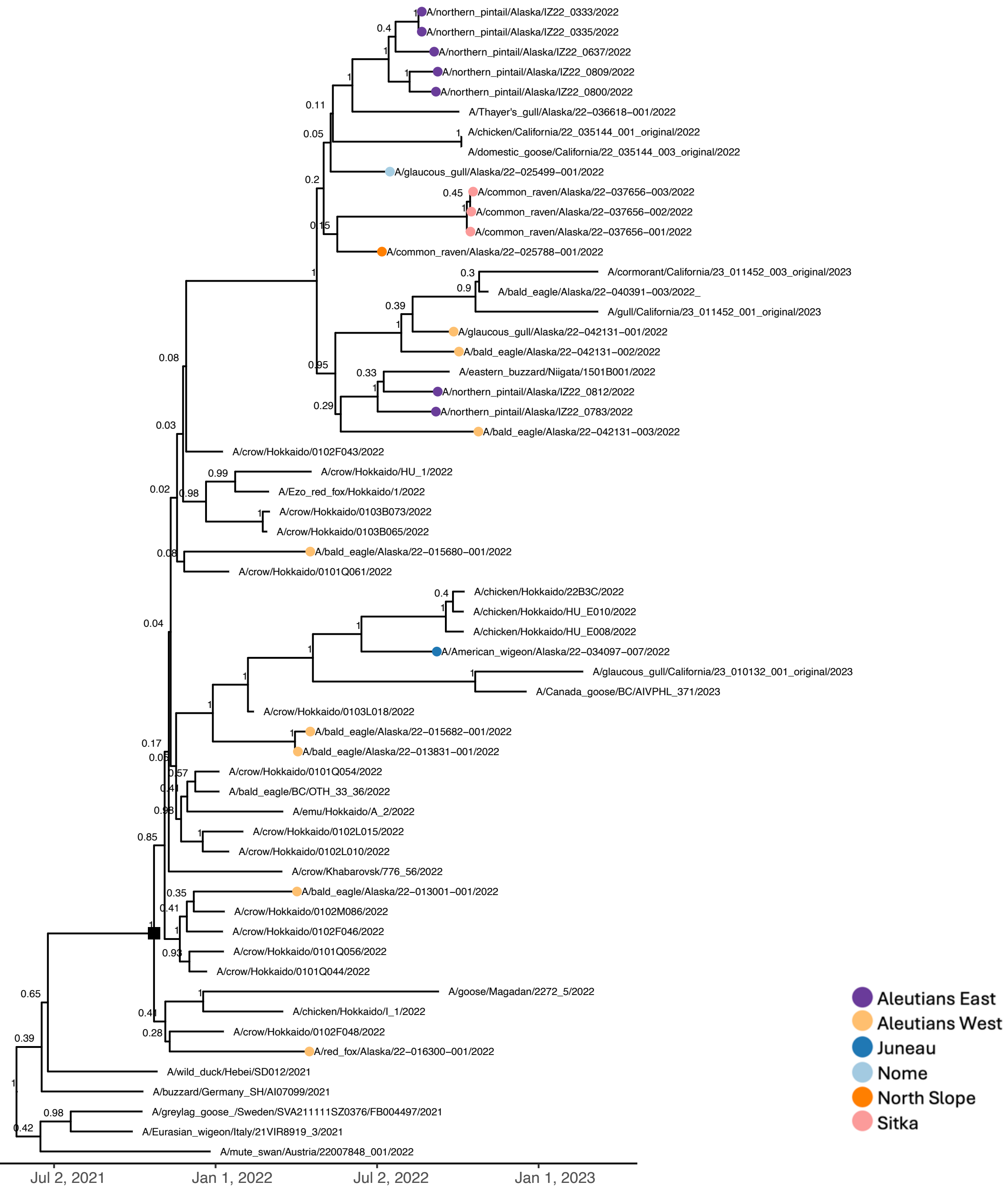

Supplementary Figure 5 Maximum clade credibility tree of Genotype A3 H5 clade 2.3.4.4b highly pathogenic avian influenza virus whole genomes from Alaska (colored tips) and elsewhere. A black square indicates the node from which the time to most recent common ancestor was estimated. Node labels indicate posterior probabilities.

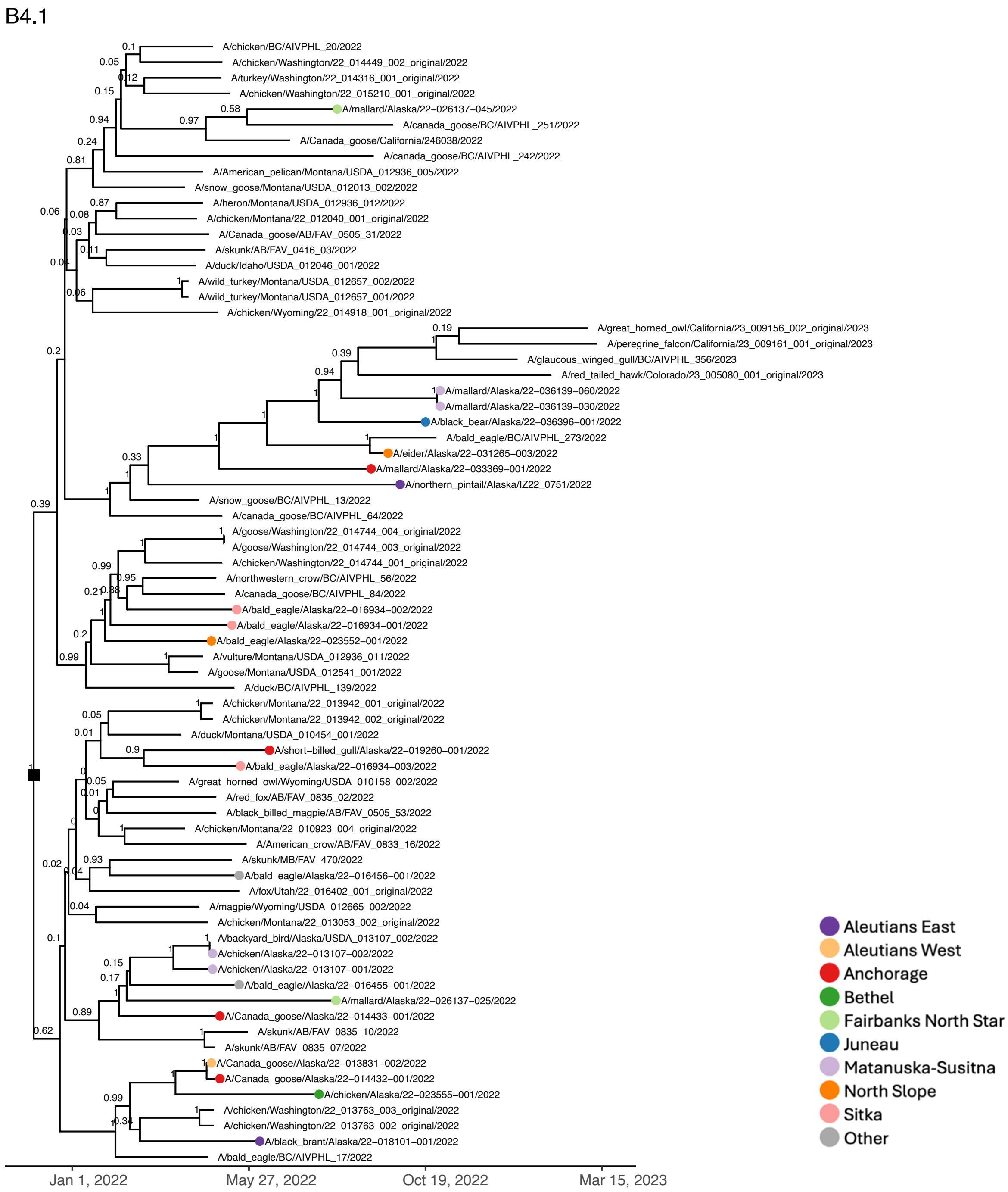

Supplementary Figure 6 Maximum clade credibility tree of Genotype B4.1 H5 clade 2.3.4.4b highly pathogenic avian influenza virus whole genomes from Alaska (colored tips) and elsewhere. A black square indicates the node from which the time to most recent common ancestor was estimated. Node labels indicate posterior probabilities.

B3.2

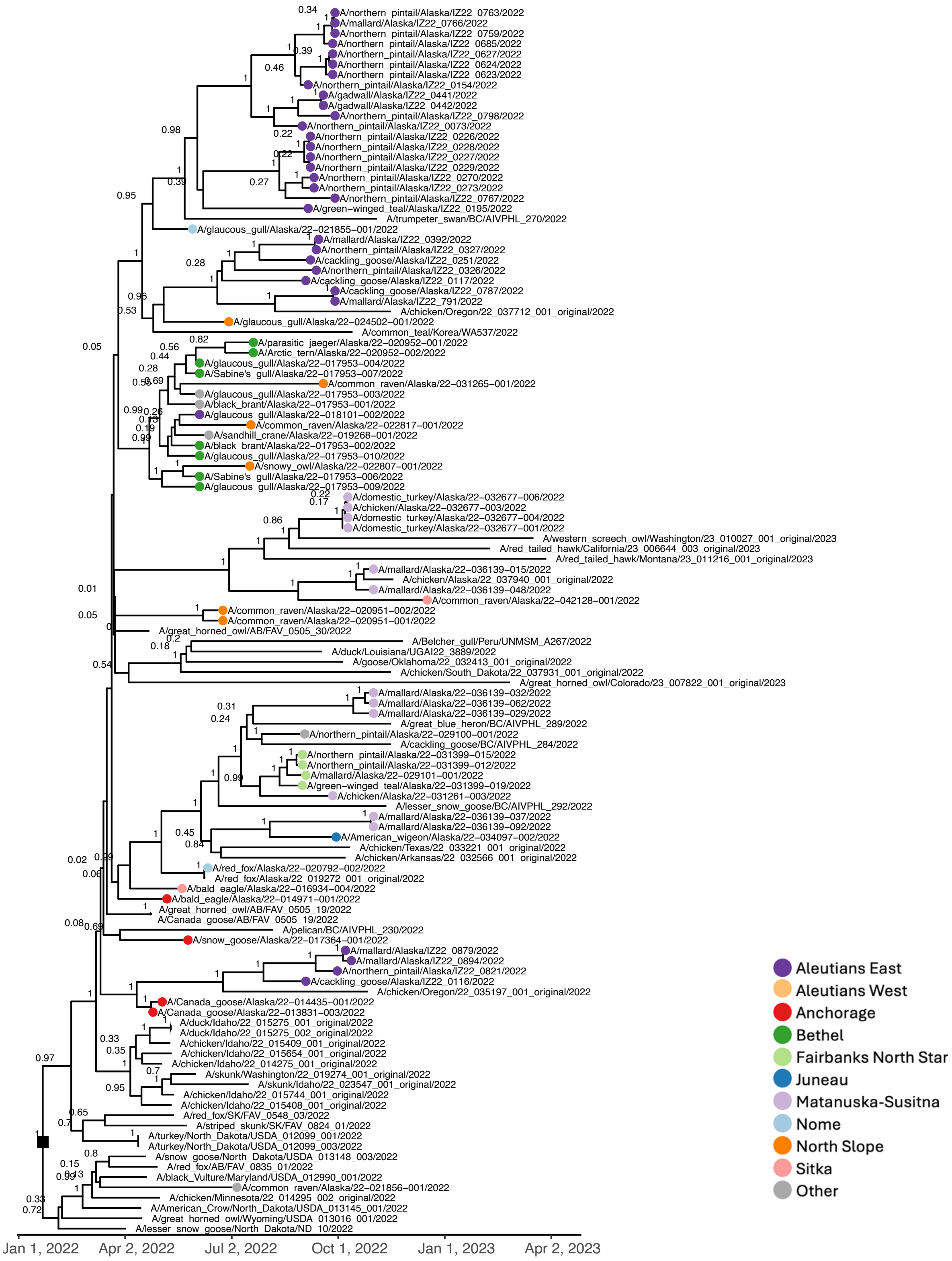

Supplementary Figure 7 Maximum clade credibility tree of Genotype B3.2 H5 clade 2.3.4.4b highly pathogenic avian influenza virus whole genomes from Alaska (colored tips)

and elsewhere. A black square indicates the node from which the time to most recent common ancestor was estimated. Node labels indicate posterior probabilities.
